# Supplementary material for: Monkeypox virus quadrivalent mRNA vaccine induces immune response and protects against vaccinia virus
Source: Signal Transduct Target Ther. 2023 Apr 28;8:172. doi: 10.1038/s41392-023-01432-5 (PMC10144886; doi:10.1038/s41392-023-01432-5)
Supplement: Supplementary file 1 — Monkeypox virus quadrivalent mRNA vaccine induces immune response and protects against vaccinia virus [file 41392_2023_1432_MOESM1_ESM.docx]

Supplementary Materials for

Monkeypox virus quadrivalent mRNA vaccine induces immune response and protects against vaccinia virus

Ye Sang^1^, Zhen Zhang^1^, Fan Liu^2^, Haitao Lu^1^, Changxiao Yu^1^, Huisheng Sun^1^, Jinrong Long^1^, Yiming Cao^1^, Jierui Mai^1^, Yiqi Miao^1^, Xin Wang^1^, Jiaxin Fang^1^, Youchun Wang^3^, Weijin Huang^2^, Jing Yang^1^ and Shengqi Wang^1^.

Correspondence to: Shengqi Wang, [sqwang@bmi.ac.cn](mailto:sqwang@bmi.ac.cn); Jing Yang, [jingyang0511@sina.com](mailto:jingyang0511@sina.com); Weijin Huang, [huangweijin@nifdc.org.cn](mailto:huangweijin@nifdc.org.cn)

**This PDF file includes:**

Figures. S1


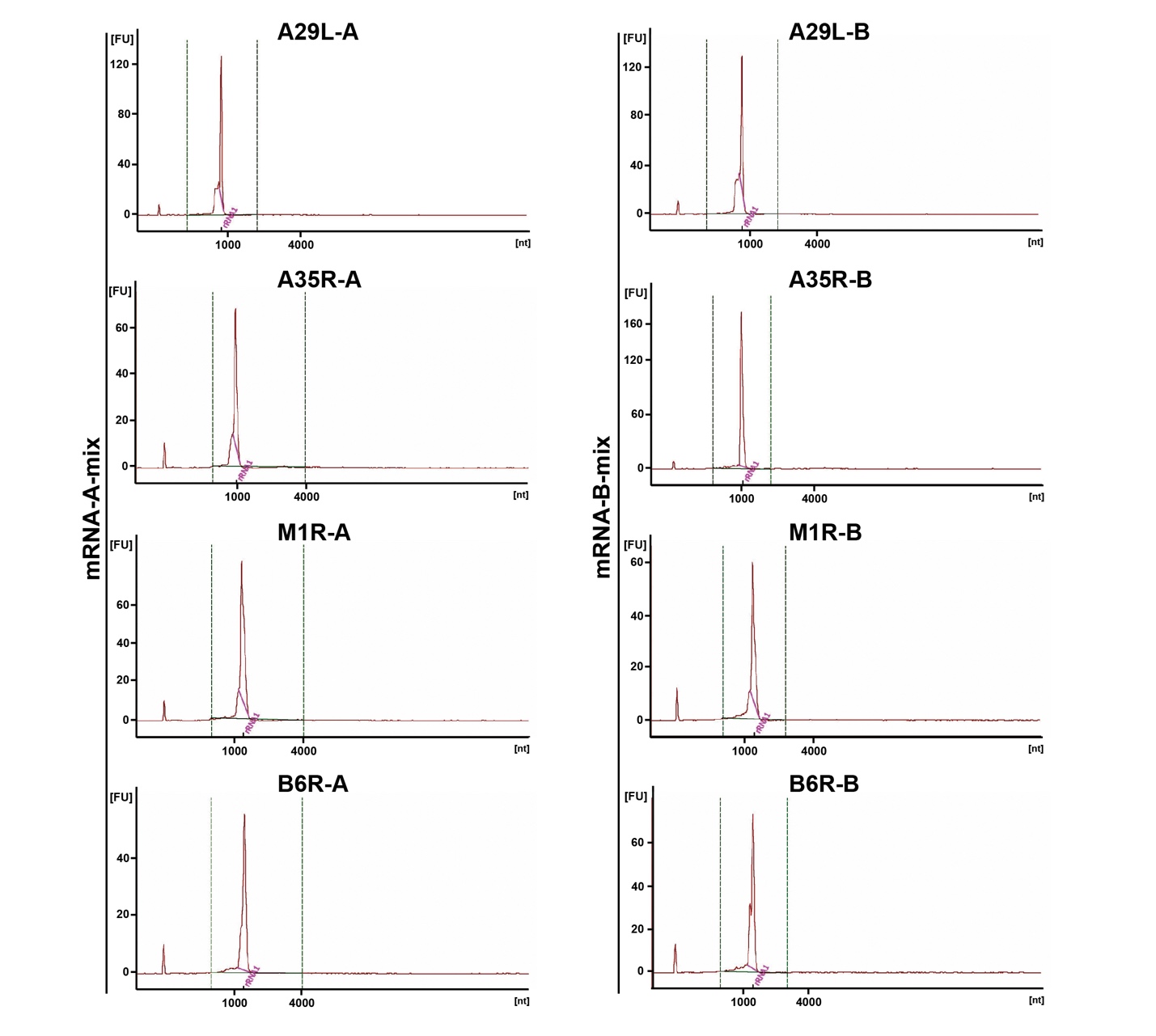


Figure. S1.

Identification of *in vitro* synthesized mRNAs. Using the Agilent 2100 Bioanalyzer system, multiple mRNAs from mRNA-A-mix and mRNA-B-mix synthesized in vitro were identified and quantified.
